# Quality of Life Outcomes After Fundoplication-Augmented One-Anastomosis Gastric Bypass: A Randomized Comparative Study

**DOI:** 10.7759/cureus.96802

**Published:** 2025-11-13

**Authors:** Mostafa Mahran, Reem Salah, Rofida Sobh, Haitham Elmaleh, Osama Fouad

**Affiliations:** 1 Upper Gastrointestinal Surgery, Homerton University Hospital, London, GBR; 2 General Surgery, Ain Shams University, Cairo, EGY; 3 Family Medicine, Ain Shams University, Cairo, EGY; 4 Upper Gastrointestinal Surgery, Ain Shams University, Cairo, EGY

**Keywords:** anti-reflux surgery, bariatric surgeries, gerd-hrql, nissen fundoplication (nf), oagb, obesity and gerd

## Abstract

Background

Gastroesophageal reflux disease (GERD) frequently persists despite weight reduction, thereby diminishing anticipated improvements in quality of life following bariatric surgery. While one-anastomosis gastric bypass (OAGB) is metabolically effective, it may not completely manage reflux symptoms. This study assessed whether the incorporation of a modified fundoplication could enhance symptom control and health-related quality of life (HRQoL) compared with OAGB alone.

Methods

A prospective randomized comparative study was conducted involving 60 patients diagnosed with obesity and GERD. Participants were randomly allocated into two groups for a follow-up period of two years: Group A underwent OAGB only, and Group B received OAGB with modified fundoplication. Outcomes were evaluated based on preoperative and postoperative upper GI and manometry findings, as well as postoperative GERD-Health-Related Quality of Life (GERD-HRQL) scores.

Results

In comparison to OAGB alone, fundoplication demonstrated superior symptom control (Visick 2.10 ± 0.71 vs. 3.57 ± 0.73; p = 0.001) and resulted in a shorter duration of PPI use (2.43 ± 0.62 vs. 3.13 ± 0.68 months; p = 0.001). The GERD-HRQL scores showed significant improvement following fundoplication at six months (48.33 ± 7.91 to 31.57 ± 3.90; p < 0.001), and this improvement was sustained up to 24 months, whereas the changes observed after OAGB alone were not statistically significant. At the 24-month mark, endoscopic evaluation revealed an intact wrap with no evidence of GERD, and manometry confirmed restoration of LES function.

Conclusions

Compared with OAGB alone, the combination of OAGB with modified fundoplication offers a distinct advantage in terms of quality of life, as evidenced by improved symptom scores, decreased reliance on proton pump inhibitors, and enhanced endoscopic and manometric control of reflux. This integrated approach represents a viable strategy for optimizing the HRQoL of patients with obesity and GERD. However, validation through larger multicenter studies with extended follow-up periods and pH impedance monitoring is necessary.

## Introduction

Obesity often occurs alongside gastroesophageal reflux disease (GERD), and both conditions significantly affect health-related quality of life (HRQoL). While bariatric surgery is effective in achieving weight loss and managing metabolic issues, the type of surgery chosen can impact reflux management and patient-reported outcomes. One-anastomosis gastric bypass (OAGB) is known for its technical ease and impressive weight loss results; however, it may lead to bile/acid reflux, gastritis, and esophagitis, which are factors that contribute to ongoing symptoms, reliance on PPIs, and reduced HRQoL [1,2].

Switching to Roux-en-Y gastric bypass is a known solution for persistent bile reflux, although it involves risks, such as internal hernias [3,4]. An alternative method involves incorporating short, loose Nissen-style fundoplication using the bypassed stomach during OAGB to strengthen the gastroesophageal junction [5,6]. This strategy seeks to prevent reflux and reestablish the high-pressure zone (HPZ) at the lower esophagus while maintaining the metabolic advantages of OAGB. This may lead to better GERD-HRQL scores, decreased reliance on medications, and increased patient satisfaction [5].

This randomized comparative study assessed the practicality, safety, and effectiveness of OAGB with modified fundoplication compared to OAGB alone, primarily focusing on quality of life outcomes for patients with obesity and GERD and/or hiatal hernia.

## Materials and methods

Study design and setting

This prospective randomized comparative study was conducted over two years at Ain Shams University Hospital. Sixty consecutive eligible adults with obesity and GERD were randomized in a 1:1 ratio to undergo either OAGB (Group A) or OAGB with a modified Nissen-style fundoplication fashioned from the excluded stomach (OAGB-MF; Group B). Randomization was performed using a sealed-envelope method. Sequentially numbered, opaque envelopes containing the group assignments were prepared by a statistician not involved in patient recruitment or surgery. This process ensured allocation concealment and minimized selection bias. The operating surgeons were aware of group allocation, but postoperative assessors and data analysts remained blinded to the treatment groups. Inclusion and exclusion criteria are summarized in Table 1. Preoperative evaluations, operative conduct, and follow-up schedules were standardized and identical across groups to minimize performance bias.

**Table 1:** Inclusion and exclusion criteria used for patient selection GERD, gastroesophageal reflux disease; PPI, proton pump inhibitor

Inclusion criteria	Exclusion criteria
BMI ≥35 kg/m² (any comorbidity) or BMI 30-34.9 kg/m² with obesity-related disease	BMI >50 kg/m²
Age 18-65 years	Age <18 or >65 years
GERD documented by upper GI endoscopy (± hiatal hernia)	Major psychiatric disorder; end-stage organ disease (hepatic, cardiac, and pulmonary)
At least one of the typical reflux symptoms (regurgitation, heartburn, belching, postprandial fullness, nausea, and inhalation signs), hiatal hernia, reflux complications (esophagitis), or PPI-refractory GERD ≥6 months	Esophageal high-grade dysplasia or cancer; major anesthetic/surgical contraindications (e.g., severe heart/respiratory failure and untreatable coagulopathy); esophageal motility disorder (markedly deficient peristalsis)

Preoperative assessment

All participants underwent standardized upper GI endoscopy and high-resolution manometry (HRM). Endoscopy assessed the presence and grade of esophagitis according to the Los Angeles classification, cardia competence, bile pooling, and the presence of a hiatus hernia. HRM was performed to exclude major motility disorders and to quantify lower esophageal sphincter (LES) function; a hypotensive LES was defined as an LES pressure <10 mmHg or an abnormal integrated relaxation pressure (IRP). Symptom burden and HRQoL were measured using the GERD-Health-Related Quality of Life (GERD-HRQL) questionnaire, a validated instrument in which higher scores indicate worse symptoms and lower HRQoL. The GERD-HRQL instrument is in the public domain and may be used without a formal license [7]. The Visick grading system is a clinician-reported outcome measure developed for evaluating postoperative symptom control following anti-reflux surgery. It is in the public domain and requires no formal permission or licensing for academic or clinical use.

Surgical interventions

All procedures were performed under general anesthesia with the patient in a split-leg reverse Trendelenburg position. Using five ports and a liver retractor, a long, narrow gastric pouch was fashioned over a 36-Fr bougie, with vertical stapling directed toward the angle of His while preserving an approximately 1 cm tissue cuff. The alimentary limb was measured 200 cm from the ligament of Treitz, a side-to-end gastrojejunostomy was created with a 30 mm linear stapler, and the enterotomy was closed with 2-0 V-Loc suture. The hiatus was dissected with intra-abdominal esophageal mobilization and repaired with interrupted non-absorbable sutures. In Group B, a short, intentionally loose 360° fundoplication (approximately 2.5-3 cm in length) was constructed from the excluded stomach and anchored to the crura to recreate a physiological HPZ. To facilitate a tension-free wrap and limit postoperative dysphagia, the posterior gastric artery and the terminal short gastric vessels were divided to ensure wide fundal mobilization.

Postoperative care and follow-up

All patients received standardized postoperative care, including intravenous fluids, multimodal analgesia, and venous thromboembolism prophylaxis, with a liquid diet introduced on postoperative day one. Follow-up comprised serial assessment of GERD-HRQL at baseline and at six, 12, and 24 months; evaluation of global symptom status using the Visick scale (grades I-IV, with lower grades indicating better outcomes); and objective testing at 24 months with endoscopy and HRM. Endoscopic evaluation recorded esophagitis grade, cardia competence, bile reflux, and small hiatus hernia recurrence. HRM quantified LES relaxation and HPZ restoration below the Z-line. The total duration of postoperative proton pump inhibitor (PPI) therapy was documented as a measure of medication exposure.

Outcomes

The primary outcome was the change in GERD-HRQL score from baseline to 6, 12, and 24 months. Secondary outcomes included Visick grade at follow-up, duration of postoperative PPI use, objective reflux control on endoscopy (esophagitis, bile reflux, cardia competence, and small hiatus hernia), and HRM (LES hypotension, IRP, and restoration of the HPZ).

Statistical analysis

The sample size was calculated to detect a minimum clinically significant difference of 5 points in GERD-HRQL scores between groups at 24 months, assuming an SD of 6, a two-sided α = 0.05, and 80% statistical power. To account for potential attrition, the required total sample was 60 patients (30 per group). Data were analyzed using IBM SPSS Statistics for Windows, Version 23.0 (Released 2015; IBM Corp., Armonk, NY, USA). Continuous variables were summarized as mean ± SD or median (IQR) according to distribution; categorical variables were summarized as counts and percentages. Normality was evaluated with Kolmogorov-Smirnov and Shapiro-Wilk tests. Between-group comparisons were performed using independent-samples t-tests for normally distributed continuous variables and Mann-Whitney U tests for non-normally distributed variables. Within-group changes over time were assessed using paired t-tests or Wilcoxon signed-rank tests, as appropriate. Categorical variables were compared using chi-square tests or Fisher’s exact tests when expected cell counts were <5. For every chi-square comparison, we report χ², degrees of freedom, two-tailed p, and Cramér’s V as the effect size; for 2 × 2 tables, df = 1, and Cramér’s V was computed as √(χ²/N) (interpreted using conventional thresholds). For t tests, we report t(df) and Cohen’s d; for Mann-Whitney and Wilcoxon tests, we report r derived from Z/√N. Where multiple time-point contrasts were examined, p-values were adjusted using Bonferroni correction. Statistical significance was set at two-sided p < 0.05.

Ethics

This study was conducted in accordance with the Declaration of Helsinki and the institutional policies and regulations of Ain Shams University. The protocol, patient information materials, and consent procedures received prior approval from the institutional review board (IRB) of Ain Shams University.

Before any study-specific procedures, all participants received clear and understandable information about the study aims, methods, potential risks and benefits, alternatives, data confidentiality, and their right to withdraw at any time without prejudice to care. Written informed consent was obtained from all the participants. Participant confidentiality was maintained through coded identifiers and secure data storage with restricted access. Adverse events and complications were monitored and managed according to institutional clinical governance pathways, and reportable events were communicated to the IRB per the local policy.

## Results

Sixty patients were equally randomized (30 per group). Age and BMI were comparable between groups with no statistically significant differences. The sex distribution showed a higher proportion of females in Group A, but this difference did not reach statistical significance (χ²(1, N = 60) = 3.77, p = 0.052, Cramér’s V = 0.25), indicating a small-to-moderate effect size and acceptable baseline balance (Table 2). Between-group comparisons of baseline comorbidities were nonsignificant (all p > 0.05; χ²(1, N = 60) range = 0.00-1.15; Cramér’s V range = 0.00-0.14), indicating small effect sizes and balanced groups (Table 3).

**Table 2:** Demographic characteristics at baseline

Variable	Group A (n = 30)	Group B (n = 30)	Test	p
Age (years), mean ± SD	45.83 ± 10.58	41.07 ± 11.03	t = 1.811	0.093
Sex, female n (%)	24 (80.0%)	17 (56.7%)	χ² = 3.774	0.052
BMI (kg/m²), mean ± SD	39.67 ± 3.17	39.50 ± 2.58	t = 0.050	0.824

**Table 3:** Obesity-related comorbidities and reflux-related diagnoses COPD, chronic obstructive pulmonary disease; GERD, gastroesophageal reflux disease

Variable	Group A (n = 30)	Group B (n = 30)	Test	p
Diabetes mellitus, n (%)	13 (43.3%)	9 (30.0%)	χ² = 1.148	0.284
Hypertension, n (%)	8 (26.7%)	7 (23.3%)	χ² = 0.089	0.766
Sleep apnea, n (%)	2 (6.7%)	2 (6.7%)	χ² = 0.000	1
Dyslipidemia, n (%)	5 (16.7%)	3 (10.0%)	χ² = 0.144	0.704
COPD, n (%)	2 (6.7%)	1 (3.3%)	χ² = 0.351	0.554
Osteoarthritis, n (%)	1 (3.3%)	0 (0.0%)	χ² = 1.017	0.313
GERD/reflux esophagitis, n (%)	15 (50.0%)	18 (60.0%)	χ² = 0.603	0.438
Hiatus hernia, n (%)	7 (23.3%)	8 (26.7%)	χ² = 0.393	0.531

Preoperative endoscopy and manometry

Baseline endoscopic findings were comparable between groups. The prevalence of esophagitis (LA A/B) did not differ significantly (χ²(1, N = 60) = 0.61, p = 0.436, Cramér’s V = 0.10), and there were no between-group differences in the occurrence of hiatus hernia (χ²(1, N = 60) = 0.09, p = 0.765, Cramér’s V = 0.04) or cardia incompetence (χ²(1, N = 60) = 1.48, p = 0.224, Cramér’s V = 0.16). All effects were small, supporting baseline comparability (Table 4). Manometric parameters were likewise similar at baseline. The proportion of patients with abnormal IRP or a hypotensive LES did not differ significantly between groups (χ²(1, N = 60) = 0.09, p = 0.767, Cramér’s V = 0.04), nor did the prevalence of a small hiatus hernia with hypotensive LES (χ²(1, N = 60) = 0.13, p = 0.723, Cramér’s V = 0.05) or a short intra-abdominal esophagus with dual pressure peaks (χ²(1, N = 60) = 0.39, p = 0.741, Cramér’s V = 0.08). Effect sizes were uniformly small, further confirming balance before intervention (Table 5).

**Table 4:** Preoperative upper GI endoscopy findings LA, Los Angeles

Finding	Group A (n = 30)	Group B (n = 30)	Test	p
Esophagitis (LA A/B), n (%)	15 (50.0%)	18 (60.0%)	χ² = 0.606	0.436
Hiatus hernia, n (%)	7 (23.3%)	8 (26.7%)	χ² = 0.089	0.765
Incompetent cardia, n (%)	8 (26.7%)	4 (13.3%)	χ² = 1.481	0.224

**Table 5:** Preoperative HRM findings HRM, high-resolution manometry; IRP, integrated relaxation pressure; LES, lower esophageal sphincter

Finding	Group A (n = 30)	Group B (n = 30)	Test	p
Abnormal IRP/hypotensive LES <10 mmHg, n (%)	23 (76.6%)	22 (73.3%)	χ² = 0.088	0.767
Small hiatus hernia <3 cm with hypotensive LES, n (%)	5 (16.67%)	4 (13.3%)	χ² = 0.125	0.723
LES <3 cm above diaphragm + dual peaks, n (%)	2 (6.6%)	4 (13.3%)	χ² = 0.389	0.741

Preoperative GERD-HRQL

Total GERD-HRQL scores were not significantly different (45.97 ± 3.93 vs. 48.33 ± 7.91; t = -1.64, p = 0.107) (Table 6).

**Table 6:** Preoperative GERD-HRQL total score GERD-HRQL, Gastroesophageal Reflux Disease-Health-Related Quality of Life

Metric	Group A (n = 30)	Group B (n = 30)	Test	p
Total score, mean ± SD (median (IQR))	45.97 ± 3.93 (50 (36-52))	48.33 ± 7.91 (55 (30-57))	t = -1.64	0.107

Postoperative global symptom status (Visick)

The Visick scores were significantly better in Group B (2.10 ± 0.71) than in Group A (3.57 ± 0.73); t = 6.244, p = 0.001. The median (IQR) was 2 (2-3) versus 4 (3-4) (Table 7).

**Table 7:** Visick score at follow-up

Metric	Group A (n = 30)	Group B (n = 30)	Test	p
Mean ± SD	3.57 ± 0.73	2.10 ± 0.71	t = 6.244	0.001
Median (IQR)	4 (3-4)	2 (2-3)	-	-

PPI exposure

Duration of postoperative PPI use was shorter in Group B (2.43 ± 0.62 months) than in Group A (3.13 ± 0.68 months); t = 4.143, p = 0.001 (Table 8).

**Table 8:** Duration of postoperative PPI therapy PPI, proton pump inhibitor

Metric	Group A (n = 30)	Group B (n = 30)	Test	p
PPI duration (months), mean ± SD	3.13 ± 0.68	2.43 ± 0.62	t = 4.143	0.001

GERD-HRQL trajectory (preoperative to 24 months)

Group A (OAGB) showed numerical decreases, without statistical significance, in the reported comparisons. Group B (OAGB + fundoplication) showed sustained early improvement for 24 months (Table 9, Table 10).

**Table 9:** GERD-HRQL over time - Group A (OAGB) GERD-HRQL, Gastroesophageal Reflux Disease-Health-Related Quality of Life; OAGB, one-anastomosis gastric bypass

Time point	Mean ± SD	Median (IQR)	Within-group test	P
Preoperative	45.97 ± 3.93	50 (36-52)	-	-
6 months	45.00 ± 3.99	46 (37-52)	t = −0.987 vs. baseline	0.328
12 months	43.57 ± 4.00	44 (36-51)	t = 0.946 vs. 6 months	0.348
24 months	43.07 ± 3.90	44 (35-50)	-	-

**Table 10:** GERD-HRQL over time - Group B (OAGB + fundoplication) GERD-HRQL, Gastroesophageal Reflux Disease-Health-Related Quality of Life; OAGB, one-anastomosis gastric bypass

Time point	Mean ± SD	Median (IQR)	Within-group test	p
Preoperative	48.33 ± 7.91	55 (30-57)	-	-
6 months	31.57 ± 3.90	32 (28-35)	t = 10.41 vs. baseline	<0.001
12 months	32.00 ± 3.57	32 (24-37)	t = -0.984 vs. 6 months	0.325
24 months	33.07 ± 3.99	33 (26-39)	-	-

Endoscopic outcomes

At 24 months, Group A showed incompetent cardia (8/30), mild-to-moderate esophagitis LA A/B (8/30), biliary esophagitis (1/30), and small hiatus hernia (<2 cm) (8/30). In Group B, intact fundal wrap with no GERD/esophagitis was seen in 28/30 patients, mild esophagitis in 5/30, incompetent cardia in 2/30, and endoscopic (not clinical) recurrence of small hiatus hernia in 2/30 (Table 11).

**Table 11:** Two-year endoscopic follow-up GERD, gastroesophageal reflux disease; HH, hiatus hernia; LA, Los Angeles

Finding	Group A (n = 30)	Group B (n = 30)
Mild-moderate esophagitis (LA A/B), n/N	8/30	5/30
Endoscopic recurrence of small HH (<2 cm), n/N	-	2/30
Intact fundal wrap; no GERD/esophagitis, n/N	-	28/30
Incompetent cardia, n/N	8/30	2/30
Biliary esophagitis, n/N	1/30	-
Small hiatus hernia (<2 cm), n/N	8/30	-

Manometric outcomes

At 24 months, Group B showed restoration of LES function: adequate LES relaxation in 28/30, increased HPZ at the LES below the Z-line in 30/30, mild IRP elevation in 6/30, and small hiatal hernia with hypotensive LES in 2/30. In Group A, incompetent cardia/abnormal IRP/hypotensive LES <10 mmHg persisted in 22/30, and small hiatus hernias with hypotensive LES persisted in 8/30 (Table 12).

**Table 12:** Two-year HRM follow-up HPZ, high-pressure zone; HRM, high-resolution manometry; IRP, integrated relaxation pressure; LES, lower esophageal sphincter

Finding	Group A (n = 30)	Group B (n = 30)
Adequate LES relaxation, n/N	-	28/30
Incompetent cardia/abnormal IRP/hypotensive LES <10 mmHg, n/N	22/30	-
Small hiatus hernia with hypotensive LES, n/N	8/30	2/30
Increase in HPZ at LES below Z-line, n/N	-	30/30
Mild IRP elevation, n/N	-	6/30

## Discussion

OAGB has become popular because of its straightforward technique, significant weight loss results, and metabolic advantages [8]. However, OAGB can lead to or exacerbate reflux in some patients, necessitating the exploration of additional anti-reflux measures [9]. Fundoplication strengthens the LES and mitigates pathological reflux [10]; nonetheless, traditional wraps, such as Nissen or Toupet, can be difficult to perform with the OAGB anatomy [11].

In response to this issue, various groups have suggested using the bypassed stomach in OAGB for fundoplication. This approach aims to create a reliable and long-lasting anti-reflux mechanism while maintaining the metabolic advantages of bypass [12].

The baseline characteristics were comparable, as there were no significant differences in age, sex, or BMI between the groups. Preoperative assessments, including preoperative endoscopy and manometry, showed no statistically significant variations in the occurrence of esophagitis, hiatus hernia, incompetent cardia, or LES motility/relaxation parameters. Although the incidence of esophagitis was higher in the fundoplication group (60% vs. 50%), this difference was not statistically significant. Preoperative GERD-HRQL scores were also similar (48.33 ± 7.91 vs. 45.97 ± 3.93; p = 0.107), indicating a balanced symptomatic burden at the start. This comparability minimizes selection bias and enhances the internal validity of the treatment-effect estimates.

These observations were consistent with those reported in the current literature. While certain cohorts reporting on OAGB with fundoplication by Werapitiya et al. [13] and Ospanov et al. [14] have noted a higher preoperative reflux burden. Collectively, this evidence supports the conclusion that our study groups were well-matched at the outset, facilitating a clearer assessment of differential postoperative trajectories.

The significant postoperative indicator was the global symptom status (Visick), which showed a preference for the fundoplication group, suggesting superior symptom management and patient satisfaction (mean 2.10 ± 0.71 vs. 3.57 ± 0.73; p = 0.001). This finding aligns with previous studies: Werapitiya et al. reported notable improvements in Visick scores following ESF (from 3.8 ± 0.39 to 1.2 ± 0.39), and Papasavas et al. found significant symptom relief after fundoplication [13,15]. In a randomized trial by Ospanov et al., the Visick scores improved in all groups, with the most significant changes observed in fundoplication variation [14].

Regarding PPI exposure, both groups experienced postoperative decreases. Nonetheless, the fundoplication group required significantly shorter PPI treatment, indicating an additional level of reflux control beyond the bypass effect alone. These conclusions were drawn from a complex evidence base. Bou Daher et al. pointed out the possibility of bile reflux following OAGB, a historical critique of early technique versions, whereas contemporary protocols and acid suppression address the acid component of reflux [16,17]. Extensive registry data show a higher long-term initiation of PPI after sleeve gastrectomy than after OAGB, reinforcing the general reflux advantage of gastric bypass setups. However, it carries the specific risk of alkaline reflux, which sometimes requires revision [18]. In this scenario, additional fundoplication may further reduce PPI reliance, consistent with our observed between-group difference (p < 0.05) and with the findings of Ospanov et al. and Soprani et al., who reported lower rates of new reflux esophagitis and symptoms when a wrap was included [14,19].

In terms of HRQoL, the GERD-HRQL scores showed significant and lasting improvement following OAGB with fundoplication, with noticeable benefits at six months, and these improvements were largely sustained at 24 months. The group that underwent OAGB alone showed numerical improvement, but these improvements were not statistically significant in the specified comparisons. These trends align with both randomized and observational studies that have shown a clinically significant reduction in symptoms after OAGB combined with fundoplication [14,15,19]. Ospanov et al. notably reported significant reductions in both mean domain and total GERD-HRQL scores (e.g., Fundo Ring OAGB from 25.2 ± 2.7 to 4.34 ± 1.3), with the ring-reinforced wrap demonstrating the most favorable HRQoL profile [14]. Alongside meta-analytic data suggesting a higher risk of GERD following OAGB than RYGB (OR 3.14; odds for developing new GERD ~6-fold) [20,21] and an increased likelihood of long-term GERD-related surgery post-OAGB [22], these findings support the need for proactive and effective reflux management when choosing OAGB in patients with existing or high-risk GERD.

Two-year objective outcomes reinforce patient-reported data. In the group with only OAGB, endoscopy often revealed an incompetent cardia, LA A/B esophagitis, biliary esophagitis, and small hiatal hernias. Conversely, the fundoplication group typically showed an intact wrap and no signs of GERD/esophagitis, with only minor instances of mild esophagitis, infrequent incompetent cardia, and occasional small clinically silent hiatal recurrence. These findings suggest that fundoplication helps maintain the integrity of the gastroesophageal junction and prevents reflux, consistent with the studies by Ospanov et al. and Soprani et al. [14,19]. Ferrer-Márquez et al.’s narrative review highlights the uncertainty and variability of bile reflux post-OAGB and recommends endoscopic monitoring, which our results support [23].

HRM conducted at the two-year mark demonstrated significant restoration of the LES in the fundoplication cohort. Most patients exhibited adequate LES relaxation, and all participants showed an increased HPZ below the Z-line. A minority of patients experienced mild IRP elevation, whereas small hiatus hernias with hypotensive LES were infrequent. Conversely, in the group that underwent OAGB alone, hypotensive LES/abnormal IRP and small hiatus hernias with hypotensive LES were common. These manometric findings suggest that the wrap effectively reestablished a functional sphincteric zone, corroborating the results of Werapitiya et al and Ospanov et al. [13,14].

The strengths of the study include randomized allocation, balanced baseline characteristics, and a comprehensive evaluation that integrates patient-reported outcomes (GERD-HRQL and Visick) with objective physiological measures (endoscopy and manometry) over a two-year period. However, the study is limited by its recruitment from a single region, modest sample size, and lack of pH-impedance monitoring, which could quantify the burden of acid versus alkaline reflux. A longer follow-up period would provide further insight into the durability of anti-reflux effects and late wrap-related adverse events, such as persistent dysphagia.

The clinical implications of this study are twofold. First, for obese patients with a clinically significant reflux burden who are otherwise suitable candidates for OAGB, the addition of concomitant fundoplication may result in enhanced HRQoL and symptom management, decreased dependence on PPIs, and improved endoscopic and manometric outcomes. Second, considering the documented risk of GERD following OAGB compared with RYGB, a selective approach that emphasizes anti-reflux augmentation when baseline GERD is present or anticipated appears to be a reasonable strategy.

## Conclusions

For patients experiencing obesity and significant reflux, the integration of a loose fundoplication utilizing the excluded stomach into OAGB demonstrates a notable enhancement in HRQoL compared with OAGB alone. This combined approach yielded lower GERD-HRQL scores (indicating improved symptom management), higher Visick grades (indicating increased patient satisfaction), and decreased reliance on PPIs while sustaining effective long-term weight loss. These patient-centered benefits are supported by consistent objective findings, such as reduced endoscopic signs of reflux and improved manometric sphincter function, suggesting durable restoration of the anti-reflux barrier. Overall, modified fundoplication offers a viable strategy to improve HRQoL in patients at risk of postoperative reflux. Larger studies with extended follow-up and pH-impedance monitoring are necessary to confirm durability and refine patient selection.
